# Nature representation in South American protected areas: country contrasts and conservation priorities

**DOI:** 10.7717/peerj.7155

**Published:** 2019-07-01

**Authors:** Germán Baldi, Santiago Schauman, Marcos Texeira, Sofía Marinaro, Osvaldo A. Martin, Patricia Gandini, Esteban G. Jobbágy

**Affiliations:** 1 Instituto de Matemática Aplicada San Luis, Universidad Nacional de San Luis & CONICET, San Luis, Argentina; 2 Departamento de Métodos Cuantitativos y Sistemas de información, Facultad de Agronomía, Universidad de Buenos Aires, Buenos Aires, Argentina; 3 Instituto de Investigaciones Fisiológicas y Ecológicas Vinculadas a la Agricultura, Universidad de Buenos Aires & CONICET, Buenos Aires, Argentina; 4 Instituto de Ecología Regional, Universidad Nacional de Tucumán & CONICET, Horco Molle, Argentina; 5 Instituto Ciencias del Ambiente, Sustentabilidad y Recursos Naturales, Universidad Nacional de la Patagonia Austral & CONICET, Puerto Deseado, Argentina

**Keywords:** Protected areas, Protection equality, Protection extent, Nature representation

## Abstract

**Background:**

South America faces strong environmental pressures as a result of agriculture and infrastructure expansion and also of demographic growth, demanding immediate action to preserve natural assets by establishing protected areas. Currently, 7.1% of the (sub)continent is under strict conservation categories (I to IV, IUCN), but the spatial distribution of these 1.3 × 10^6^ km^2^ is poorly understood. We evaluated the representation of nature within the networks of protected areas, map conservation priorities and assess demographic, economic or geopolitical causes of existing protection patterns.

**Methods:**

We characterized nature representation by looking at two components: the extent and the equality of protection. The first refers to the fraction of territory under protection, while the second refers to the homogeneity in the distribution along natural conditions of this protected fraction. We characterized natural conditions by either 113 biogeographical units (specifically, ecoregions) or a series of limited and significant climatic, topographic and edaphic traits. We analyzed representation every ten years since 1960 at national and continental levels. In the physical approach, histograms allowed us to map the degree of conservation priorities. Finally, we ranked the importance of different economic or geopolitical variables driving the observed distributions with a random forest technique.

**Results:**

Nature representation varied across countries in spite of its priority in conservation agendas. In Brazil, Peru and Argentina there are still natural conditions with no formal protection, while in Bolivia and Venezuela, protected areas incorporate the natural diversity in a more balanced manner. As protected networks have increased their extent, so did their equality across and within countries over time. Our maps revealed as top continental priorities the southern temperate, subhumid and fertile lowland environments, and other country-specific areas. Protection extent was generally driven by a low population density and isolation, while other variables like distance to frontiers, were relevant only locally (e.g., in Argentina).

**Discussion:**

Our description of the spatial distribution of protected areas can help societies and governments to improve the allocation of conservation efforts. We identified the main limitations that future conservation efforts will face, as protection was generally driven by the opportunities provided by low population density and isolation. From a methodological perspective, the physical approach reveals new properties of protection and provides tools to explore nature representation at different spatial, temporal and conceptual levels, complementing the traditional ones based on biodiversity or biogeographical attributes.

## Introduction

Over the last three decades, most South American countries have undergone an unprecedented expansion of cultivated lands, infrastructure and urban areas (Pacheco, 2012). These changes reflect and lead to national economic growth. However, they also pose negative local to global environmental impacts, mainly associated with the loss of biodiversity and with the decrease in the provision of multiple ecosystem services (Carreño, Frank & Viglizzo, 2012). Under the dominant economic logic and mainly due to the still great availability of resources and low population densities, this region will probably maintain or increase its role as a global supplier of raw materials (Alexandratos & Bruinsma, 2012). In this sense, protected areas stand as one of the most effective tools to protect nature in all its forms in the long term (Hoekstra et al., 2005).

**Figure 1:**
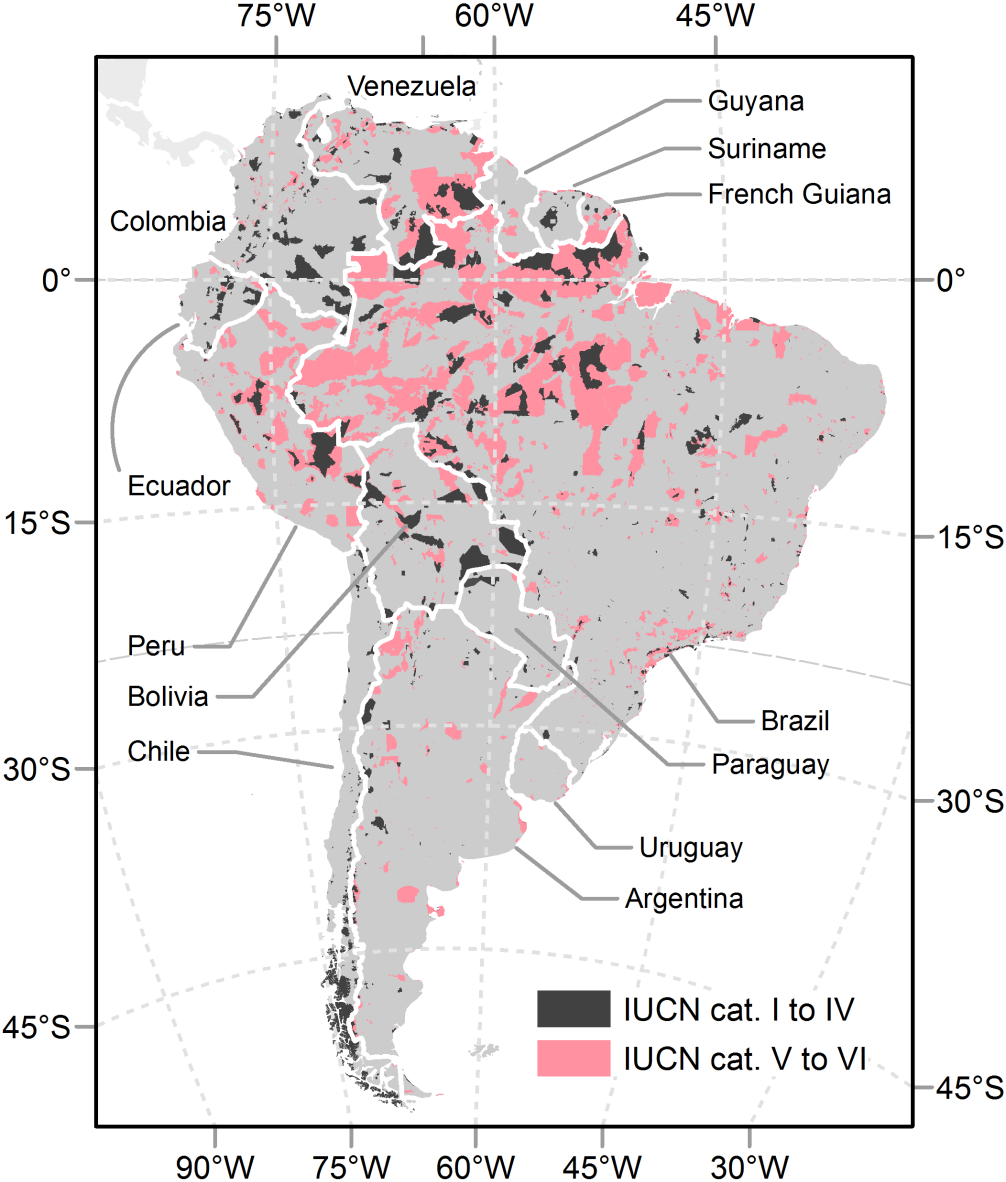
Protected areas in South America. Protected areas from the “World Database on Protected Areas” (IUCN & UNEP-WCMC, 2016). Only terrestrial areas categorized as I to IV (IUCN, 1994) were considered in analyses.

Past conservation efforts of individuals and conservation agencies have resulted in a globally significant increase in protected areas from year to year (Chape et al., 2005; Watson et al., 2014). In the early 20th century, South American countries followed the seminal ideas of North America and Europe, which emphasized preserving iconic landscape features (Wirth, 1962). Almost a century later, more than one thousand protected territories exist under diverse legal titles (e.g., national parks, reserves, or monuments). They encompass 7.1% of the South American continental surface (1.3 × 10^6^ km^2^, almost the size of Peru) under strict conservation categories (I to IV, IUCN, 1994), a fraction slightly above the global value (Fig. 1 and Table 1). However, this growing area does not yet provide an adequate representation of natural conditions at a continental level (Baldi et al., 2017). Two factors are likely to account for this. The first is the complex interplay of cultural motivations that lead to effective protection, like guarding economically valuable assets or biodiversity hotspots (McNeely, Harrison & Dingwall, 1994; Watson et al., 2014). These motivations have been of variable strength through history and across territories. The second factor is associated with the limitations that different human forces (e.g., cultivation) impose over motivations, which ultimately drive protection to areas that face little human intervention and may have comparatively low opportunity-costs, at least at the time of their establishment (Joppa & Pfaff, 2009; Baldi et al., 2017).

**Table 1:** South American networks of protected areas. General description of the networks of protected areas according to the “World Database on Protected Areas”. Annual Release from October 2016 (IUCN & UNEP-WCMC, 2016). Territories outside South America are depicted to contextualize continental values.

**Territory**	**Area (1,000 km**^**2**^**)**	**Area under protection, cat. IUCN I–IV (1,000 km**^**2**^**)**	**Protection extent, cat. IUCN I–IV (%)**	**Number of protection units, cat.IUCN I–IV**	**Area under protection, cat. IUCN I–VI (1,000 km**^**2**^**)**	**Protection extent, cat. IUCN I–VI (%)**	**Number of protection units, cat.IUCN I–VI**	**First protected area (yr)**
Argentina	2,793	64	2.3	131	243	8.8	360	1934
Bolivia	1,099	178	16.2	53	258	23.8	195	1939
Brazil	8,515	478	5.6	676	2,464	29.2	2,066	1914
Chile	756	139	18.3	126	129	18.4	149	1907
Colombia	1,142	135	11.8	86	156	13.9	600	1977
Ecuador	284	37	13	19	50	20	48	1959
French Guiana (FG)	91	4	4.9	26	44	53	29	1979
Guayanas (FG+GU+SU)	470	20	4.3	50	81	18.6	83	1929
Guyana (GU)	215	0.61	0.28	3	19	8.9	25	1929
Paraguay	407	15	3.6	26	22	5.4	37	1906
Peru	1,285	85	6.6	28	370	28.8	212	1966
Suriname (SU)	164	15	9.2	21	19	13	29	1961
Uruguay	176	0.15	0.09	4	6	3.4	11	2006
Venezuela	916	140	15.2	45	383	42.5	106	1937
All South American countries	18,321	1,296	7.1	1,294	4,244	23.2	3,950	1907
Australia & New Zealand	7,958	703	8.8	14,163				1840
Canada & USA	19,379	1,847	9.5	9,193				1872
Bhutan	39.9	16.5	41.2	10				1966
Costa Rica	51.3	9	17.5	61				1955
Kenya & Tanzania	1,534	197	12.8	123				1905
Scandinavia & Finland	1,261	113	9.0	6,451				1909
Globe	134,650	8,188	6.1	82,942				1838

Recently, the search for nature representation has been intensively promoted by conservation agencies. Representation implies that any network of protected areas has to preserve a targeted area extent and, at the same time, has to sample all the natural conditions and at all levels of life organization in a balanced manner (Margules & Pressey, 2000). Furthermore, both the structure of nature and its functioning may last over time and not be altered by human interventions. In this line, the parties of the 2010 Aichi Convention on Biological Diversity (CBD) negotiated different conservation targets (SCBD, 2010). Among them, the first clause of the Target 11 stipulated that at least 17% of terrestrial areas and inland waters needed to be included within protected networks by 2020. The second clause stipulated that networks needed to be ecologically representative, though no protection equality value was set (except the 17% for all units). Although these targets do not consider local constraints and have no scientifically defined endpoints (Woodley et al., 2012), they are fundamental in stressing a global policy on how nature needs to be protected due to its intrinsic, non-utilitarian value.

A wide collection of studies has evaluated the effectiveness of existing networks to represent and encompass nature considering the protection extent of biogeographical units, like ecological systems, ecoregions, biomes or realms (McNeely, Harrison & Dingwall, 1994; Aycrigg et al., 2013), and countries or continents (Chape et al., 2005; Barr et al., 2011). Juffe-Bignoli et al. (2014) stated that only 43% of the terrestrial ecoregions worldwide meet the 17% goal. In South America, though a strong diversification of protected networks has taken place since the 1960s, most biogeographical units are far below the Aichi Target 11, especially coastal areas and those areas with strong economic activity (Elbers, 2011). Notable exceptions are the Southern Andes temperate and subpolar forests (Pliscoff & Fuentes-Castillo, 2011) and the Amazonian moist forests (Jenkins & Joppa, 2009).

Current conservation literature on representation reveals two theoretical and methodological biases. First, only a few studies address the extent of protection along continuous physical gradients like temperature or altitude (e.g., Kamei & Nakagoshi, 2006; Joppa & Pfaff, 2009; Baldi et al., 2017), and most describe it on the basis of predefined biogeographical units (e.g., ecoregions, biomes). This bias on how nature is conceived and measured is notable, given the indissoluble relationship between the physical environment and the structure and functioning of the ecosystems, and given the intrinsic value of the physical environment as a constituent part of nature (Schimper et al., 1903; Holdridge, 1947; Del Grosso et al., 2008; Huston, 2012). Second, few studies analyze whether protected areas are equally distributed in space (across physical gradients and biogeographical units) within a territory (e.g., Barr et al., 2011; Kuempel, Chauvenet & Possingham, 2016; Baldi et al., 2017). Given these biases, we propose a joint exploration of the two complementary dimensions of representation (i.e., extent and equality), considering natural conditions by means of predefined biogeographical units (a traditional, biocentric approach) and physical variables (a new, complementary and customizable approach). We aim, therefore, to provide a more comprehensive picture of any conservation status. This information would make it possible to evaluate efficiencies and advances in representation under alternative settings of protected area networks.

In this paper, we first characterize the protection extent and equality of natural conditions within terrestrial protected areas in South America explicitly designated for nature protection—i.e., those categorized as I–IV under the International Union for Conservation of Nature guidelines (IUCN, 1994). Second, we explore the relationship between extent and equality among and within countries every 10 years from 1960 to 2016. Third, we map conservation priorities according to the current spatial distribution of protection along physical gradients. Fourth, we relate the current spatial distribution of protection to human factors (demographic, economic or geopolitical).

**Table 2:** List of physical and human-related variables. List of physical (i.e., climatic, topographic or edaphic) and human-related (i.e., demographic, economic or geopolitical) variables used to evaluate the distribution of protected areas.

**#**	**Variable**	Calculation and source	**Use**
1	Temperature	Mean annual values in °C, from the “Ten Minute Climatology data base” (crudata.uea.ac.uk) (New et al., 2002), representing averaged monthly figures for the 1961–1990 period. Spatial resolution: 10 arc-min	To explore the equality dimension of representativeness
2	Precipitation	Annual precipitation in mm. Same source and spatial resolution as temperature	
3	Elevation	Mean values from the “Shuttle Radar Topography Mission” (earthdata.nasa.gov, SRTM) digital elevation model (USGS, 2004). Spatial resolution: 90 m. In km above sea level	
4	Terrain slope	Same source and spatial resolution as elevation. In degrees	
5	Soil fertility	Mean values in the cell of the top-soil total exchangeable bases (TEB, 0–30 cm), in cmolc * kg^−1^. From ISRIC-WISE - Global data set of derived soil properties (daac.ornl.gov, v.3.0) (Batjes, 2006). Spatial resolution: 30 arc-min	
6	Tourism attractiveness	Ratio of total “Flickr” photos (https://www.flickr.com) to total population counts in the cell, in photos * inh^−1^. Modified from the “World touristiness map” (http://www.bluemoon.ee). Flickr photos were downloaded in April 2017 and processed with Python v.2.7. Population came from the source used in variable #8	To explain the current geographical patterns
7	Distance to frontiers	Mean Euclidean distance in km from vector data from “Natural Earth” (http://www.naturalearthdata.com). Cartographic scale: 1:50 m	
8	Population	Total inhabitants in the cell from the “Gridded Population of the World v.3 (GPWv3): Population Grids” for the years 1990–1995 (sedac.ciesin.columbia.edu) (CIESIN-CIAT, 2015). Spatial resolution: 2.5 arc-min	
9	Distance to roads	Representing the isolation of the territory. Minimum Euclidean distance in km from vector data from the OpenStreetMap (http://www.openstreetmap.org, OSM) data (as of 2017-04-09), considering ‘primary’, ‘secondary’, ‘tertiary’ and ‘trunk’ classes. Venezuela data come from “Vialidad de Venezuela” (IGVSB, 2017), considering similar classes from the OSM. Minimum values would represent the human context of the surrounds of protected areas	
10	Cropland suitability	Land suitability for low input level rain-fed crops, considering cereals, soybean, and oil palm (http://www.fao.org/nr/gaez) (FAO/IIASA, 2011). Calculated as the maximum suitability of the included species, per pixel (unitless). Spatial resolution: 5 arc-min	

## Methods

### Data sources

Data of protected areas for South America came from the “World Database on Protected Areas”, Annual Release October 2016 (IUCN & UNEP-WCMC, 2016). For all analyses, we considered only terrestrial areas explicitly designated for nature protection, i.e., IUCN categories I to IV (1994). We used all polygonal units (*n* = 1,294) and created circular buffers for those units with only point locations and estimated extents. Regarding these points, we only considered units whose buffered polygons did not intersect existing protected area polygons (*n* = 18). In this database, most protected areas in Bolivia, Ecuador and Paraguay were labeled under the IUCN class “Not Reported”, which could lead to an underestimation of their national figures. In this sense, exclusively for these three countries, we considered previous UNEP-WCMC categorizations. In order to achieve the first three objectives (i.e., to calculate protection extent and equality over time, and map priorities), we evaluated the spatial and temporal distribution of protected areas across natural conditions, characterized by (i) 113 biogeographical units from the Olson et al. (2001) “Terrestrial ecoregions of the world” database and (ii) the combination of 5 continuous physical variables representing main climatic, topographic and edaphic traits of a territory (variables #1 to #5, Table 2). For the third objective, biogeographical data was discarded from the exploration. In order to achieve the fourth objective (i.e., to assess human drivers of protection extent), we evaluated the current spatial distribution of protected areas across human factors related to different motivations of conservation (McNeely, Harrison & Dingwall, 1994; Watson et al., 2014; Baldi et al., 2017) (variables #6 to #10,Table 2). Specifically, “tourism attractiveness” quantifies the influence of aesthetic/recreational values of a territory in the implementation of protected areas. The “distance to frontiers” depicts the importance of protection close to international borders, conceiving these as territories where it is necessary to assert national sovereignty in a peaceful manner. The last three variables, “population”, “distance to roads” and “cropland suitability”, quantify protection in territories that have a low economic value for traditional and profitable land uses (Baldi et al., 2017).

### Sampling procedure

We explored the spatial and temporal distribution of protected areas at national and continental levels (Fig. 1). The Guayanas, i.e., French Guiana, Guyana and Suriname, were considered a single unit due to their relatively small size and physical homogeneity. We sampled data following two complementary approaches. In the first approach, protection, physical and human-related data were summarized in a grid of 55,414 cells of 0.1° × 0.1°. In the second approach, only protection data were summarized in the above mentioned biogeographical units. The first approach allowed us to analyze representation and map conservation priorities on a physical basis (first three objectives) and to assess human drivers of conservation (fourth objective). The second approach allowed us to analyze both dimensions of representation on a biogeographical basis (first two objectives).

Compared to other sampling approaches in which each protected area is treated as a single sample unit, gridding offers the advantages of (i) providing a unified spatial resolution for all variables, (ii) encompassing the full range of physical and human factors, (iii) avoiding the averaging of these conditions within very large protected areas and (iv) providing a representation of the context of protected areas by characterizing the full grid cell in which they are embedded (97.5% of the cells include unprotected conditions).

### Data analysis

**Figure 2:**
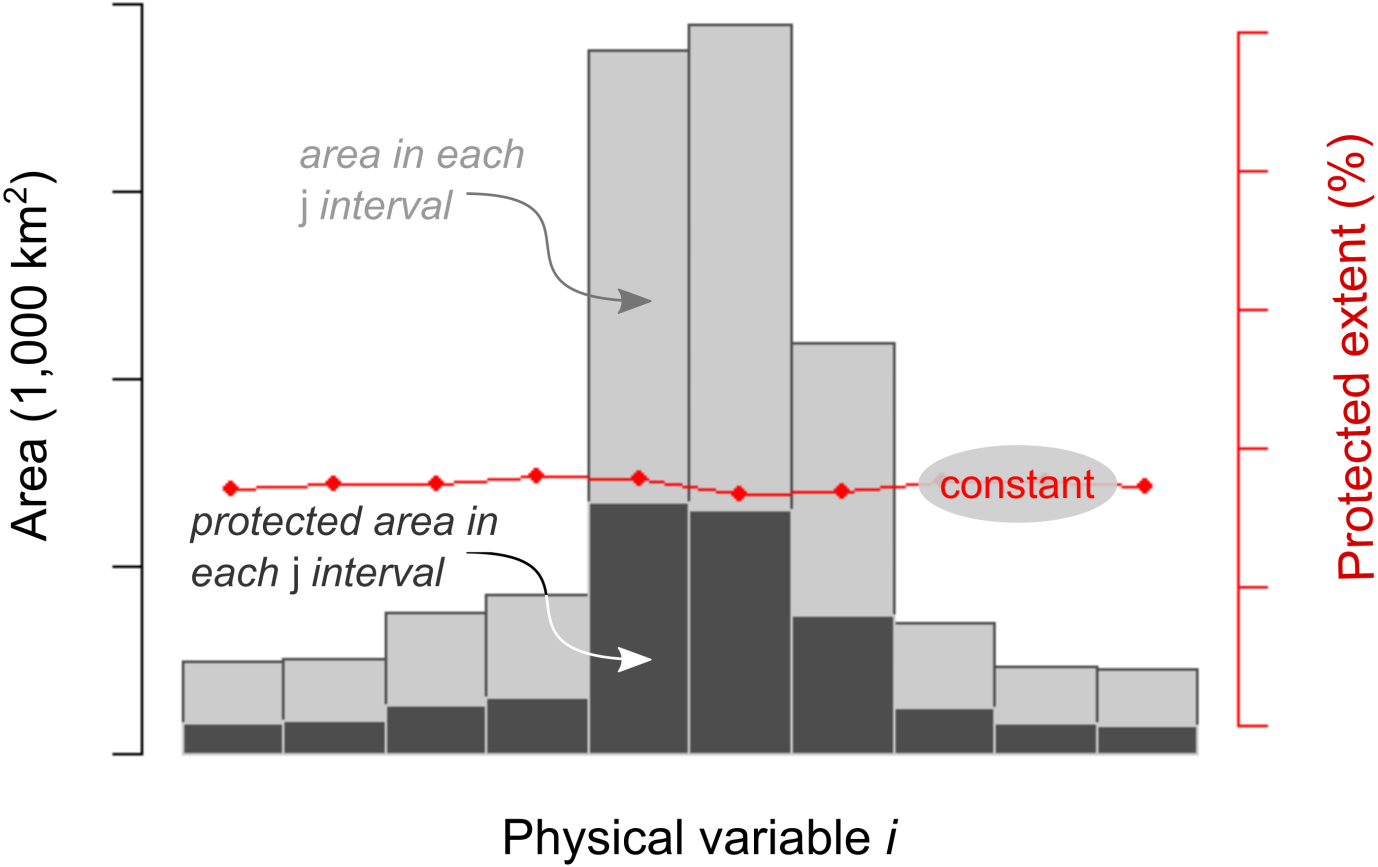
Expected protection pattern according to the representation motivation. Model of a completely balanced protected area network along an *i* physical continuous physical gradient (e.g., temperature). The encircled text refers to the expected and tested balanced behavior. Three measurements are shown in each interval of the histogram: the absolute area extent (light gray bars), the absolute area extent under protection (dark gray bars) and the relative area extent under protection (red dots –PEx_ij_, and lines). PEx_ij_ is the single variable used to calculate protection equality.

For the first sampling (gridded) approach we generated 840 histograms—i.e., 12 territories * 10 continuous variables * 7 temporal periods–, containing three sets of information: (i) the absolute extent (in 1,000 km^2^) of each class of the *i* continuous variable, (ii) the absolute extent under protection status (in 1,000 km^2^) of each *j* class (interval in the histograms) of the *i* continuous variable and (iii) the relative extent under protection PEx_ij_ (in %) of the class *j* of the *i* continuous variable (Fig. 2). In those cells shared by two or more countries, PEx_ij_ values corresponded exclusively to a targeted country in calculations. All histograms had a predefined number of 10 bins or classes, regardless of the variability of the *i* continuous variable in the territory (i.e., a country or the entire continent). In order to avoid long tails in the histograms, lower and upper *j* classes were grouped using the percentile values 0.025 and 0.975 of the *i* continuous variable. We conducted the statistical analyses corresponding to the first three objectives with the PEx_ij_ values; the two remaining sets of information were shown only for descriptive purposes.

In order to explore protection equality, we analyzed the spatial and temporal distribution of PEx_ij_ values for each territory along the gradients of the 5 *i* continuous physical variables (Table 2) by means of the Gini coefficient (G_i_) (Barr et al., 2011; Chauvenet et al., 2017) using the following equation from *ineq* R package (Zeileis, 2015):

\begin{eqnarray*}{G}_{i}= \frac{2}{n} \frac{\sum _{j}^{n}PE{x}_{ij}j}{\sum _{j}^{n}PE{x}_{ij}} - \frac{n+1}{n} \end{eqnarray*}

where *n* is the number of classes along the *i* continuous physical variables (*n* = 10). As G_i_ measures the inequality among values of a frequency distribution, we calculated a measure of equality by taking 1 −G, hereafter called G_i_′. If all PEx_ij_ values are equal for all *j* classes of the *i* continuous physical variable, G_i_′ achieves a maximum equality of value 1, regardless of the PEx_ij_. If the PEx_i_ of *j* class is different from 0, and the PEx_i_ of remaining *j* classes are 0, G_i_′ achieves a minimum equality of value 0.

We achieved a single G′ value by territory by averaging the five G_i_′ values. However, in this averaging we reduced the effects of multicollinearity between continuous physical variables by eliminating an *i* variable from the analysis if the module of its correlation coefficient with the *i+1* variable resulted greater than a predefined value of 0.55, according to a Kendall’s *τ* non-parametric test (Whittaker, 1987) (Fig. S1). In the end, we also calculated the G′ to assess the stability of the standardized binning method considering a variable number of classes following the Sturges approach (1926). For the second sampling approach (biogeographical) a G′ was also calculated, but PEx values were calculated on each ecoregion within each territory.

With a purpose similar to that of Jantke et al. (2019), and in order to map the priorities of conservation (Pr, in %) based exclusively on the *i* continuous physical variables, we analyzed the difference (in percentage) to a condition of absolute protection (100% of the area) using the following the equation:

\begin{eqnarray*}{\mathrm{Pr}}_{k}= \frac{1}{m} \sum _{i=1}^{m}(100\text{%}-{\mathrm{PEx}}_{i}) \end{eqnarray*}

where *k* represents the grid cell in each territory and *m* the number of continuous physical variables. Again, we removed *i* variables with a —Kendall’s *τ*— >0.55. In this sense, those grid cells that have low PEx_i_ values will have a large difference to a 100% protection condition and therefore, will achieve a higher Pr. We chose the condition of absolute protection equal to 100% in order to have in all cases positive Pr values.

Finally, we built histograms to characterize the behavior of the protection extent along gradients of human factors (variables #6 to #10, Table 2) in terms of shape and sign, but not to rank the relative importance of these variables as causal drivers. In this sense, for the fourth objective, we applied a random forest algorithm (Breiman, 2001) for the current protected area. This ensemble learning method estimates the variable importance (VI) by looking at how much the mean square error (MSE) increases when the out-of-bag data (OOB) for that variable is permuted while all others are left unchanged (Liaw & Wiener, 2002). The allocated VI can differ substantially with the selection of number of trees to grow (*ntree*), the minimum size of the terminal nodes (*nodesize*), or the number of input variables at each split (*mtry*) (Grömping, 2009). We chose those *mtry* values that minimize the OOB-MSE of the model (*ntree* = 500 and *nodesize* = 1). We calculated a VI variability by means of a bootstrapping technique, performing the resampling 1000 times. The VI was used here with an explanatory and interpretative rather than predictive aim.

All calculations were run in RStudio v. 1.0.143 (RStudio Team, 2018) (packages foreign, ggplot2, ggrepel, gridExtra, ineq, lattice, png, Segmented) and Python v.2.7 (packages Scikit-learn, Numpy) (Pedregosa et al., 2011; Van der Walt, Colbert & Varoquaux, 2011).

## Results

**Figure 3:**
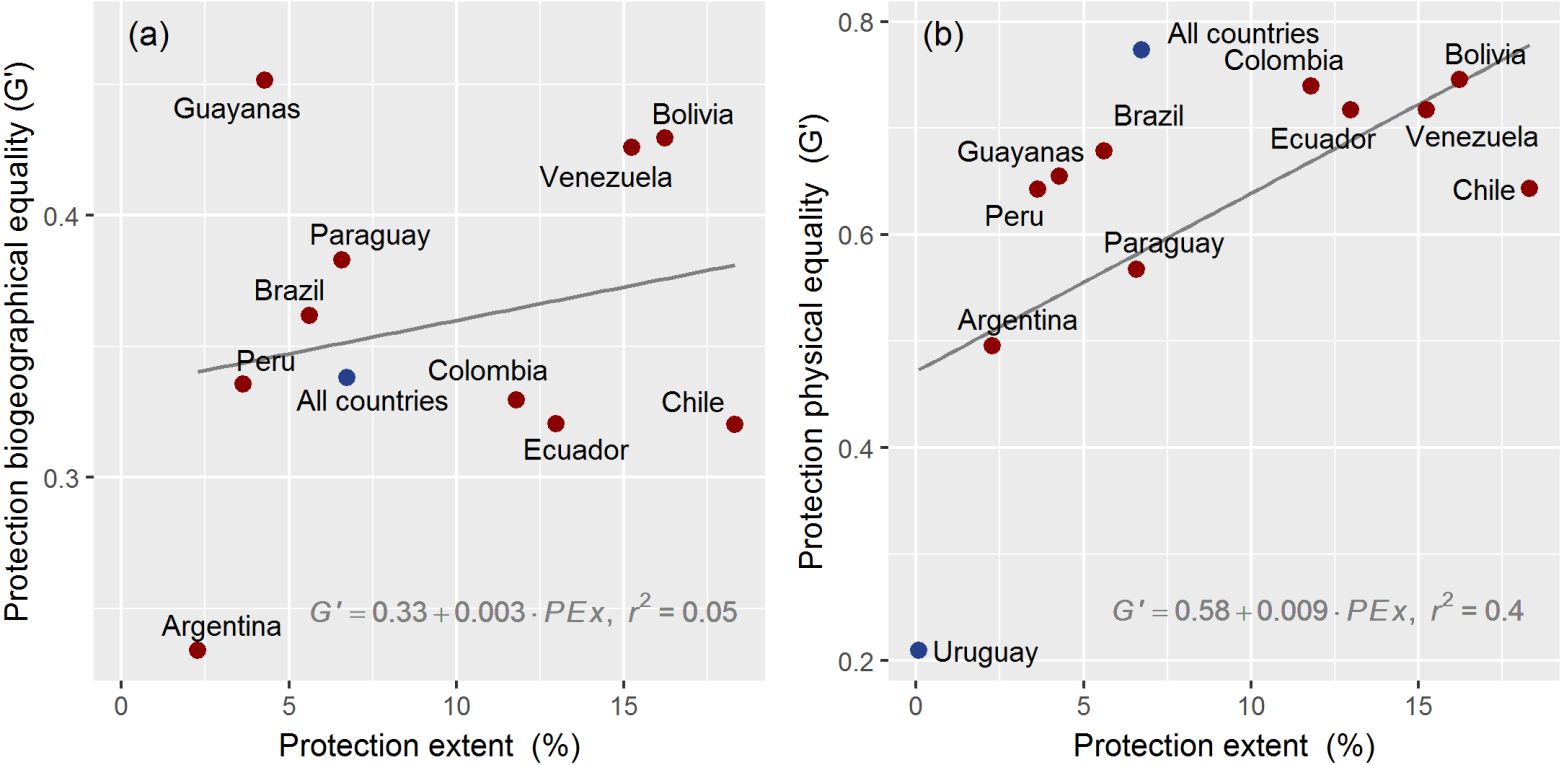
Current nature representation. Relationship between current protection extent (%) and equality (G’) in South America. In (A) equality is calculated on the basis of biogeographical units (i.e., ecoregions) and in (B) equality is calculated on the basis of physical continuous variables. Continental and Uruguayan results (in blue) were not used for linear regressions. As Uruguay has only one ecoregion, no equality value is quantified in (A).

South American countries strongly contrasted in the equality of protection of their natural conditions (Fig. 3). Chile, Bolivia and Venezuela ranked highest regarding protected extent (Table 1). The Guayanas, Bolivia and Venezuela achieved the highest equality values when natural conditions were represented by biogeographical units (G′ > 0.40). The difference in equality between the Guayanas (leading country) and Argentina (last country in the ranking) as measured by G′, was 1.9 (Fig. 3A). Bolivia, Colombia, Venezuela and Ecuador achieved the top of the ranking when natural conditions were represented by physical variables (G′ > 0.72) (Fig. 3B). Bolivia and Colombia showed an equality of physical conditions 3.5 times higher than Uruguay and 1.5 times higher than Argentina (the second country in a bottom-up ranking).

When considering biogeographical units, we found no relationship between the two analyzed dimensions of protection (*G*′ = 0.33 + 0.003 PEx, *r*^2^ = 0.05). For example, the Guayanas and Argentina reached contrasting equality values despite both territories having a low and relatively similar protected extent (4.3% and 2.3%, Table 1), while Chile and Peru reached a similar equality (G′ ≈ 0.32) with a different protected extent. In contrast, the linear relationship between the two protection variables was characterized by a weak slope and a high one coefficient of determination (*G*′ = 0.58 + 0.09 PEx, *r*^2^ = 0.4) when we considered physical variables. Notably, Chile, in spite of having the highest protection extent among countries (18.3%, Table 1), exhibited a low-to-medium equality value (*G*′ = 0.64). No notable changes occurred in the above-mentioned patterns when a different binning method was applied in the generation of histograms (Fig. S2).

**Figure 4:**
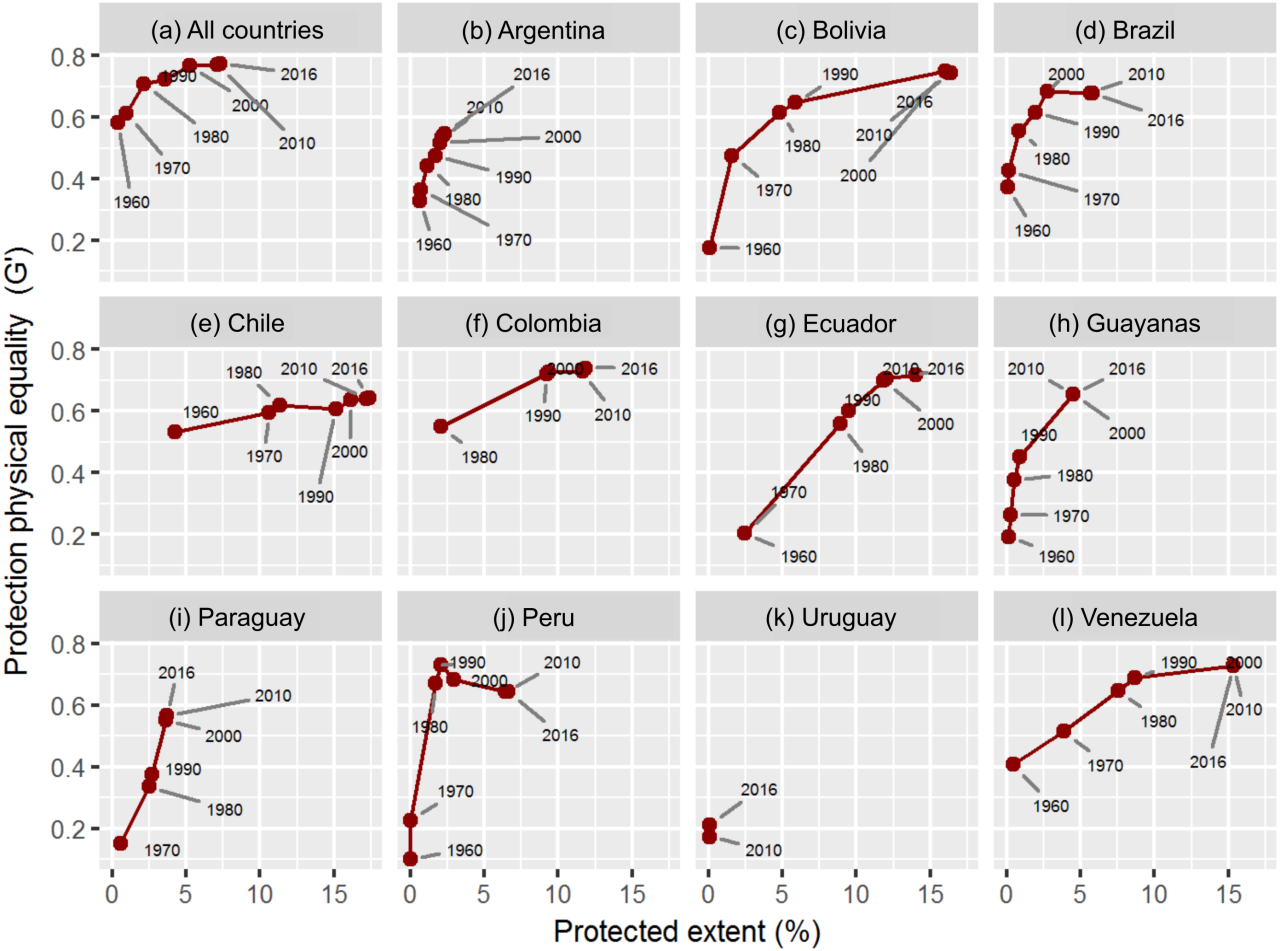
Evolution of nature representation based on physical variables. Temporal evolution of the relationship between protection extent (%) and equality (G′) in South America. Equality is calculated on the basis of physical continuous variables. Each dot indicates the end point of a temporal period except for the 1960 one, which indicates the data before 1960, inclusively. (A) All countries, (B) Argentina, (C) Bolivia, (D) Brazil, (E) Chile, (F) Colombia, (G) Ecuador, (H) Guayanas, (I) Paraguay, (J) Peru, (K) Uruguay, (L) Venezuela.

The above-described patterns were unrelated to the date in which each country created its first protected area, as shown by the new and old networks of Colombia and Venezuela (1977 vs. 1937, Table 1). Historically, as the extent of protected networks increased so did their equality (Fig. 4). However, we did not identify a typical or synchronized trend of representation in the last 5 decades, even though most countries reached their maximum equality between 1990 and 2000. The individual behavior of countries was characterized either by a general parallel increase in both extent and equality (i.e., small in Argentina, or large in Ecuador), a steady increase in extent but not in equality (i.e., in Chile), or an increase in the extent which eventually reduces equality. Additionally, decade-to-decade changes in both dimensions of representation did not follow a pattern of acceleration or stabilization. We found that the rates of increase in equality generally diminished in comparison with the rate of creation of new protected areas, with notable exceptions in Argentina and Paraguay during the 1980–2010 period (Fig. S3). The biogeographical approach to calculating equality offered a similar historical pattern, but with large differences among countries (Fig. S4).

**Figure 5:**
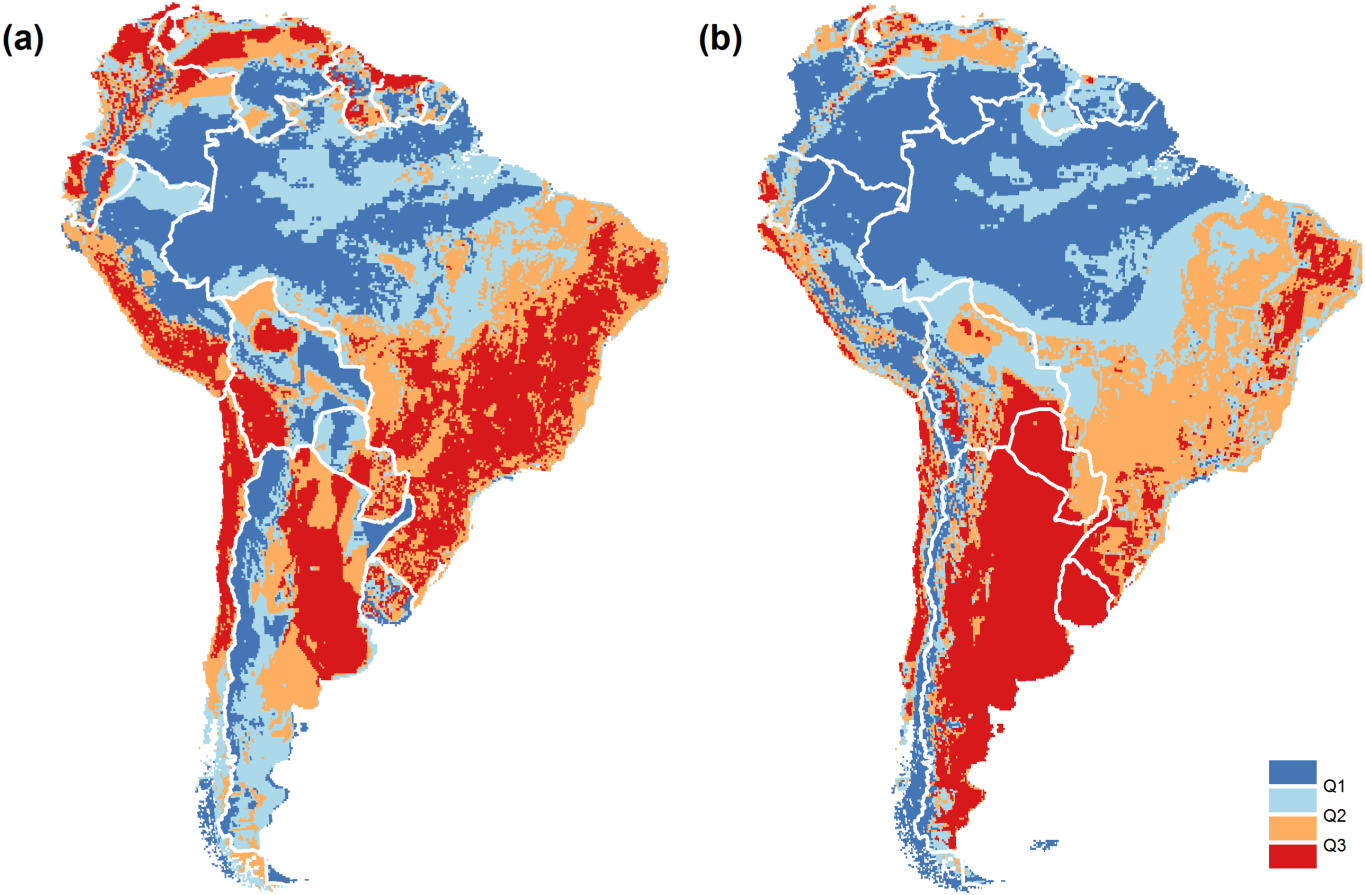
Conservation priorities. (A) National conservation priorities (Pr) in South America according to the current spatial distribution of protection extent along physical gradients. (B) Same as previous, but considering the continent as a single unit of analysis. Pr data is classified into quartiles (Q), i.e., each class encompasses a quarter of the grid cells. Red represents the highest priority, blue the lowest. White lines represent national divisions. Detailed maps are presented in Fig. S5.

Based on the current spatial distribution of protection along physical gradients, maps revealed continuities and discontinuities of conservation priority among countries (Fig. 5A and Figs. S5 and S6). When priority areas match known ecoregions, we mention them due to their popularity in the scientific literature. The hot, humid plains of the Llanos and the surrounding broadleaf forests in Colombia and Venezuela are an example of a transboundary natural system that deserves more attention. The Ecuadorian and Peruvian Andes and the subhumid highlands of the Atlantic forest in Argentina, Brazil and Paraguay, are examples of divergent conservation needs. Generally, most national priorities coincide with temperate subhumid areas originally covered by grasslands and hot arid to semiarid areas originally covered by deserts, shrublands, savannas and dry forests. A detailed description of the national priorities is depicted in Table S1. Continental priorities were the subtropical and temperate plains and plateaus of Argentina and Uruguay; and the coastal dry areas of the Pacific coast, northeastern Brazil and northwestern Venezuela (Fig. 5B and Fig. S5). The South Andes and the humid tropical lowlands of the Amazonian basin achieved notable high protection levels, with numerous, and relatively very large protected areas (e.g., Jaú National Park in Brazil and Bernardo O’Higgins National Park in Chile). Additionally, from the G′ values for each variable (Fig. S6), we found that elevation and terrain slope were the variables with the highest equality of protection at the continental level (G′= 0.88 for both variables) and at the national level (average G′= 0.66 and G′ = 0.71, respectively). Conversely, temperature and precipitation were the variables with the lowest equality of protection at the continental level (G′ = 0.69 for both variables) while precipitation and soil fertility were the ones at the national level (average G′ = 0.57 for both variables).

**Figure 6:**
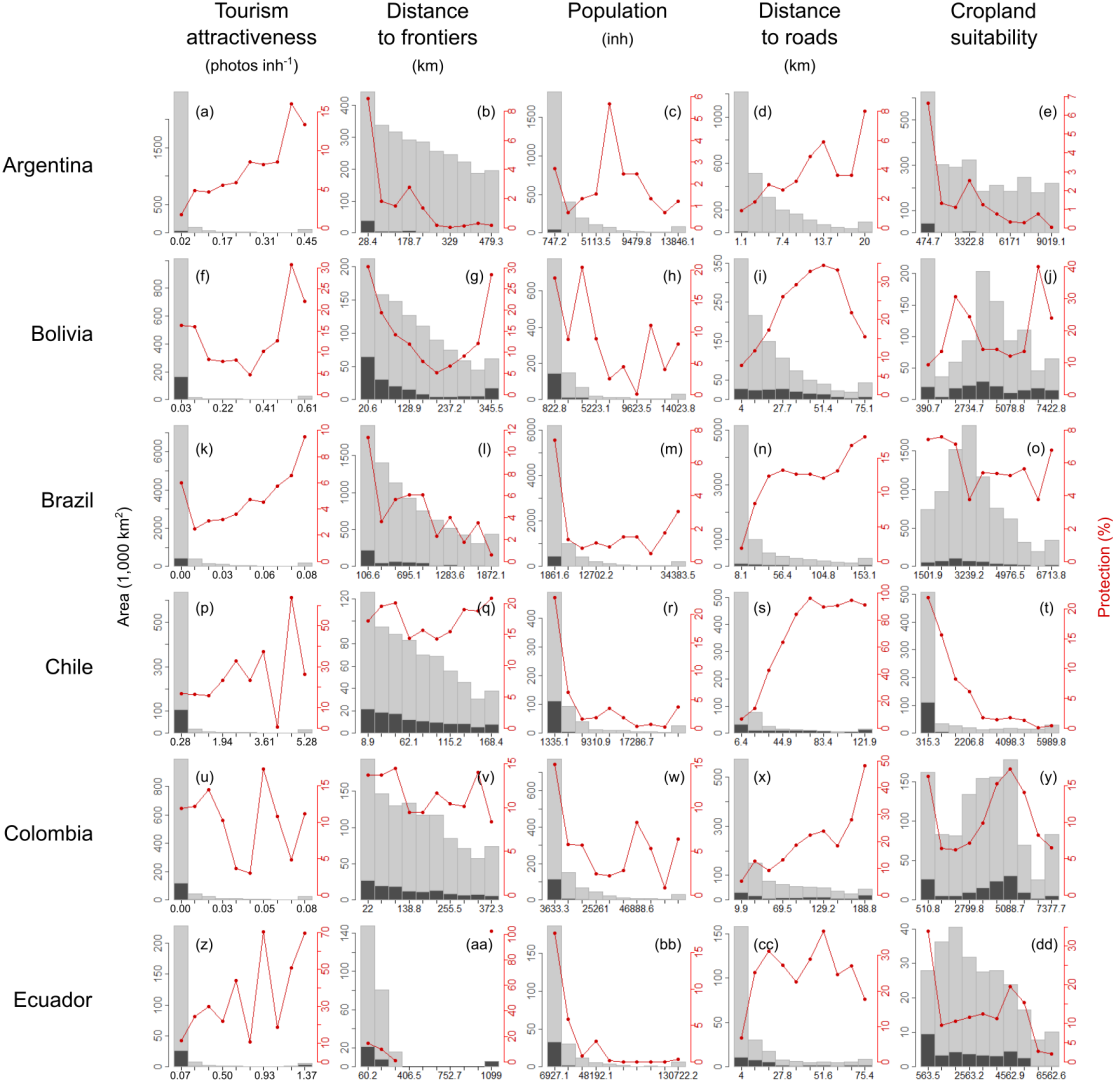
Human-related drivers of protected areas. Current spatial distribution of protection extent along human-related gradients (demographic, economic, or geopolitical) in South America. See graphic explanations in Fig. 2. Lower and upper *j* classes were grouped using the percentile values 0.025 and 0.975 of the *i* continuous variable. Argentina (A–E), Bolivia (F–J), Brazil (K–O), Chile (P–T), Colombia (U–Y), Ecuador (Z–DD).

**Figure 7:**
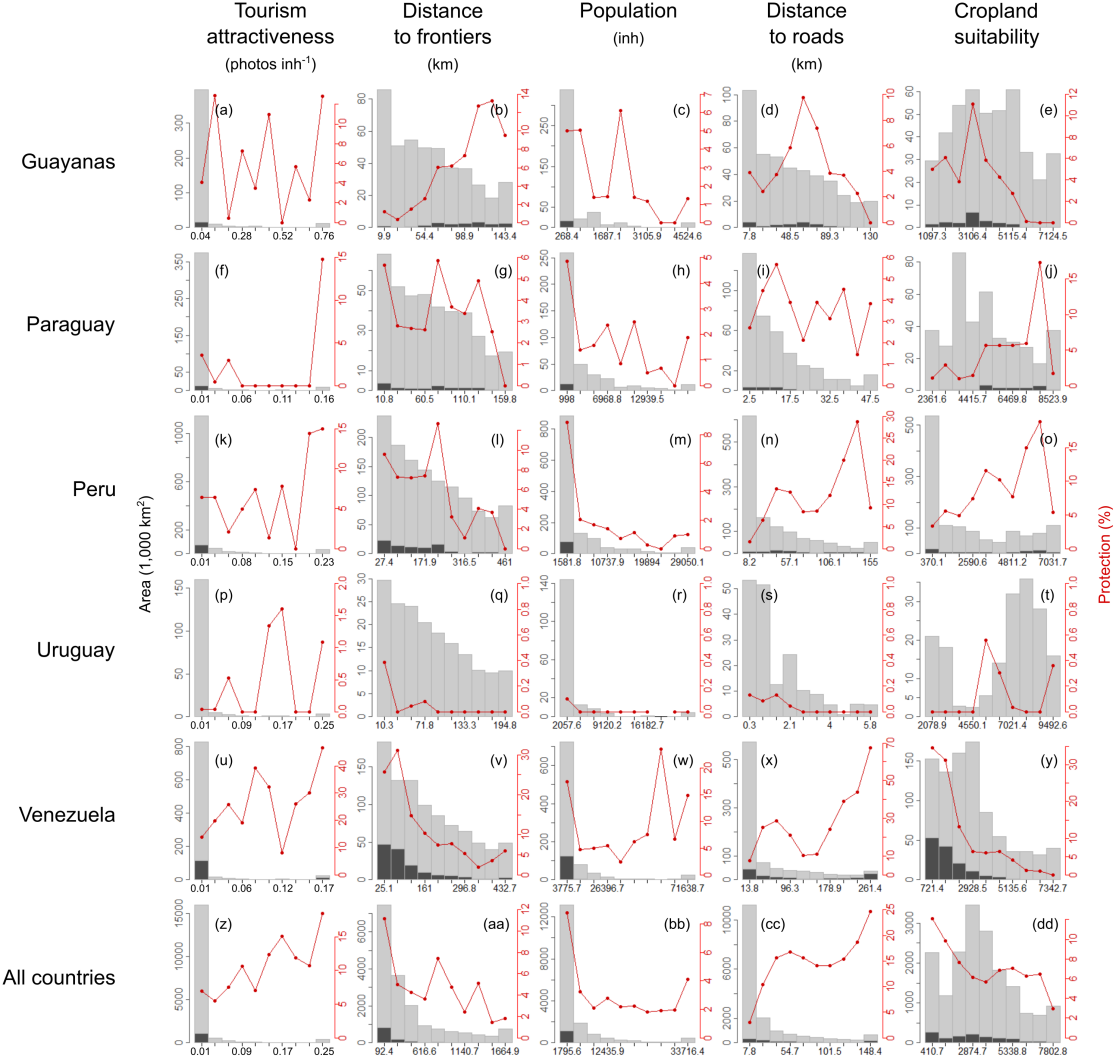
Human-related drivers of protected areas. Current spatial distribution of protection extent along human-related gradients (demographic, economic, or geopolitical) in South America. See graphic explanations in Fig. 2. Lower and upper *j* classes were grouped using the percentile values 0.025 and 0.975 of the *i* continuous variable. Guayanas (A–E), Paraguay (F–J), Peru (K–O), Uruguay (P–T), Venezuela (U–Y), All South American countries (Z–DD).

The current spatial distribution of protected areas was related to the historical conservation limitations that demographic, economic, or geopolitical forces imposed (Figs. 6 and 7, Figs. S5 and S6). According to the random forest analysis, population was the most important human factor explaining the spatial distribution of protected areas, followed by distance to roads, distance to frontiers, cropland suitability and tourism attractiveness (from VI = 27.1 to VI = 10.7, by averaging countries; Table 3). Protected areas were preferentially allocated in sparsely populated areas, especially in Peru, where its importance was 20% higher than in Paraguay (the next country on the list). The farther a territory was from a road, the greater the protection level. In Chile, the distance to roads had the highest relevance, with a magnitude 1.5 times higher than in Colombia, the next country in the ranking (VI = 39 ± 1.2 and 26.9 ± 0.7, respectively). Protected areas were closer to international frontiers, especially in Argentina and Brazil (VI = 24.9 ± 0.7 and 24.6 ± 0.3, respectively). The effect of cropland suitability on protection differed among countries with positive or negative relationships. Remarkably, in Argentina and Chile, cropland suitability had a strong negative relationship with the spatial distribution of protected areas, but relatively small importance according to the random forest. Finally, tourism attractiveness seems to have played a role driving conservation in Uruguay (VI = 31.2 ± 5.3) and in Argentina, Chile and Ecuador (VI ≈ 14).

**Table 3:** Variable importance according to a random forest. Relative importance and standard deviation (in % MSE) of the five human-related factors by territory according to the random forest analysis. The variable importance is depicted by the increase in the mean square error when the out-of-bag data for a variable is permuted while all others are left unchanged.

**Territory**	**Tourism attractiveness**	**Distance to frontiers**	**Population**	**Distance to roads**	**Cropland suitability**
Argentina	14.6 ± 0.9	24.9 ± 0.7	27.8 ± 0.8	18.1 ± 0.9	14.6 ± 0.6
Bolivia	6.3 ± 0.4	22.0 ± 0.6	29.0 ± 0.7	20.9 ± 0.5	21.9 ± 0.5
Brazil	3.9 ± 0.2	24.6 ± 0.3	29.0 ± 0.4	21.9 ± 0.3	20.5 ± 0.3
Chile	13.4 ± 0.5	15.2 ± 0.5	25.3 ± 0.7	39.0 ± 1.2	7.1 ± 0.4
Colombia	5.7 ± 0.3	21.0 ± 0.5	24.9 ± 0.5	26.9 ± 0.7	21.5 ± 0.6
Ecuador	14.1 ± 1.3	20.7 ± 1.3	23.6 ± 1.7	19.6 ± 1.1	22.1 ± 1.7
Guayanas	8.4 ± 1.3	22.0 ± 1.1	27.5 ± 1.7	20.8 ± 0.9	21.3 ± 0.9
Peru	5.9 ± 0.7	18.3 ± 0.7	35.9 ± 1.1	18.1 ± 0.8	21.9 ± 0.7
Paraguay	6.2 ± 1.9	19.4 ± 1.3	29.9 ± 3.2	22.7 ± 1.2	21.8 ± 1.5
Uruguay	31.2 ± 5.3	20.2 ± 2.6	19.2 ± 1.9	6.2 ± 1.4	23.2 ± 3.0
Venezuela	8.4 ± 0.7	18.9 ± 0.6	25.8 ± 0.7	22.8 ± 0.7	24.1 ± 0.7
Country average	10.7	20.7	27.1	21.5	20.0
All South American countries	6.1 ± 0.2	21.6 ± 0.1	27.8 ± 0.2	24.2 ± 0.2	20.4 ± 0.1

## Discussion

Representative coverage of the physical and biological nature of South America within protected areas is far from being achieved considering areas explicitly designated for protection (i.e., IUCN I to IV—1994), in spite of being an explicit priority in national and international agendas (e.g., SCBD, 2010). Extensive and physically and ecologically diverse countries like Brazil, Peru and Argentina, which accounted for almost 70% of the continental area, still underrepresent a large fraction of their nature (G′ <0.68 when considering physical variables). Colombia, Bolivia, Venezuela and Ecuador performed better when considering the two protection variables for the physical approach, surpassing the 12% of land protection and a similar and relatively high equality value (G′ ≈ 0.73 by applying the same approach). According to Barr et al.’s ranking (2011), Bolivia, Venezuela and Ecuador achieved an equality of protection 25% lower than Costa Rica, or 50% lower than Bhutan (countries renowned for their pioneering conservation efforts). As a whole, South America slightly surpasses the global protection extent (7.1% vs. 6.1%, Table 1), but is still 10% under the Aichi Target 11. Regarding protection equality, the continent occupies a low to intermediate position, below Europe, Central Asia and Sub-Saharan Africa (Baldi et al., 2017).

Our results suggest that the chance of balancing natural conditions increases by expanding the extent of protected networks, as previously described by Kuempel, Chauvenet & Possingham (2016) upon a biogeographical basis. The positive relationship among protection metrics arises both in the spatial comparison between countries (Fig. 3B) and in the temporal comparison within countries (Fig. 4). Our models propose a general stabilization or even a decrease in the equality value as networks expand (Fig. 4 and Fig. S3). A tentative explanation for this result is that each new protected area potentially encompasses a wider range of natural conditions (Marinaro, Grau & Aráoz, 2012). On the one hand, Chile illustrates the stabilization behavior, probably due to the difficulties to compensate, through the incorporation of unconsidered natural conditions, a network in which large tracts of subpolar forests were initially incorporated (near half of today’s Magellanic ecoregion is under public protection). On the other hand, the historical trends of representation in Peru and Brazil exemplify the equality decline behavior, as a result of the incorporation of large or numerous protected areas over already well-represented conditions. In Peru but not in Brazil, Kuempel, Chauvenet & Possingham (2016) showed that the expansion of protected areas in the 1993–2004 period had an aggregated spatial pattern rather than a random one. Together, these two countries hold almost three-quarters of the iconic Amazon rainforest, a natural system that has attracted strong protection efforts in the second half of the 20th century (Peres & Terborgh, 1995). According to Jenkins & Joppa (2009), this region accounted for most of the global protected area expansion of the 2000 decade.

Multiple reasons may explain why countries that share strong cultural and historical traits perceive and effectively protect their natural resources in such different ways (Table 1 and Fig. 3). Recently, we suggested that geographical differences in the distribution of protected areas were likely to reflect the interactions among policies and economy (e.g., economic context), social organization (e.g., ONG and philanthropic actions) and moral considerations (e.g., religion) (Baldi et al., 2017). In this sense, some narratives and quantitative studies describing the roots of South American protected areas highlight the effects of the (asynchronous) occurrence of financial surpluses and the alternation between government types (Barker, 1980; Pauchard & Villarroel, 2002; Marinaro, Grau & Aráoz, 2012; Leal, 2017). According to these studies, autocratic governments and young democracies prior to 1970 prioritized aesthetic/recreational values, geopolitical hotspots or potential forest production, while from the 1970s onwards, the effective protection of emerging representation and biological conservation values was tied to financial surpluses. We showed that early and modern protected areas were established preferentially in isolated or sparsely populated territories with reduced agricultural capacity and close to international borders (Table 3 and Figs. 6 and 7) (Baldi et al., 2017). In this regard, setting aside new land to preserve biodiversity is likely to succeed in areas of comparatively low opportunity costs for economic activities, but collides in areas with agriculture, forestry and mining.

If intentions to enhance the representation of natural conditions had prevailed and had received adequate financial support in the last four or five decades, national administrations would not have been able to reverse the strong initial bias in the spatial distribution of protected areas (e.g., subpolar forests in Chile), as shown by our temporal analyses (Fig. 4 and Fig. S4) and by trend and correlative analyses in Kuempel, Chauvenet & Possingham (2016). Modern protected areas tend to be of small size and follow isolated rather than systematic conservation efforts (Marinaro, Grau & Aráoz, 2012; Kuempel, Chauvenet & Possingham, 2016). Given the unique temporal trends in the representation of each country, we think that historical conservation patterns could be attributed to national rather than to international policies. Radeloff et al. (2013) and Kuempel, Chauvenet & Possingham (2016) support this observation by showing that advances in the protection extent and equality were unsynchronized among countries and occurred during brief periods named “hot moments”. Some of these hot moments occurred in the first years of national networks, as in the case of Argentina in the thirties/fifties (Marinaro, Grau & Aráoz, 2012), Chile in the sixties (Pauchard & Villarroel, 2002), and Colombia in the seventies (Leal, 2017).

We found that from 2010 (Conference of the CBD Parties) to October 2016, only 32,000 km^2^ of new protected areas were added to the continental network under categories I to IV. With less than a year left to reach the Aichi target, we provide maps that help conservation agencies focus their efforts by following the criterion of representation (Fig. 5 and Fig. S5). See Jantke et al. (2019) for further information about spatially-explicit metrics of representation. The temperate to subtropical grassy-to-woody plains of eastern Argentina and Uruguay (coincident with the Pampas and Chaco ecoregions) account for the largest territory of high continental and national priority. It is worth mentioning that even though the Pampas and Chaco have diverged in their land use history, they share threats and challenges to conservation (Brown et al., 2006; Overbeck et al., 2015). The relatively low protection of the natural conditions in these two countries also results from the fact that, in South America, tropical conditions are more abundant than temperate/cold conditions. Near the tropics, national priorities coincide with dry systems, which have recently caught the attention of scientists and conservationists due to their unappreciated biodiversity, rapid changes and high agricultural potential (Grau et al., 2014). In contrast, disagreements between continental and national maps reveal that priorities in one country might entail more strenuous efforts if these conditions are uncommon in it, but well represented in neighboring countries. Likewise, well represented national conditions can still deserve further conservation efforts if those are underrepresented in neighboring countries. This can be the case of the well-protected subhumid highlands in Argentina vs. the situation in Brazil and Paraguay (Atlantic forest ecoregion) (Huang et al., 2009; Henriques, 2011).

Spatially-explicit priorities like the ones we mapped can inform conservation agencies where to focus future efforts. However, priority maps do not consider real-world limitations, namely: the persistence of natural vegetation, its structural and functional condition and its level of isolation, land tenure or acquisition costs. Natural vegetation has been totally removed or profoundly transformed in large tracts of the continent, with urban settlements and croplands occupying 22% of its area (Ellis & Ramankutty, 2008). These same restrictions must have faced the promoters of the first protected areas. As an example, the intense use of the territory in the Argentinean and Uruguayan Pampas (Colomé, 2009) or the Brazilian Atlantic forests (Henriques, 2011) precedes the great transformations that took place in the last decades.

We should acknowledge that the role of additional protected areas not considered in our analyses may greatly alter results, as shown in Fig. 1 and Table 1. Previous assessments for Argentina (APN, 2007), Bolivia (Larrea-Alcázar et al., 2016), Brazil (Rylands & Brandon, 2005), Chile (Pliscoff & Fuentes-Castillo, 2011), Guyana (Bicknell et al., 2017) and Peru (Shanee et al., 2017) highlight the importance of these unconsidered protected areas in the achievement of targets and agreements. Aichi Target 11 did not explicitly specify under which categories this specific area should be encompassed (Woodley et al., 2012). Although IUCN categories V and VI have a dual role in promoting the preservation of biodiversity and the local economic welfare, a statutory limit for resource exploitation is not stipulated (Shafer, 2015). Besides, these governmental, communal or private protected areas potentially lack formal protection and management and have uncertain conservation objectives and long-term capabilities (Shafer, 2015). The effect of considering all categories becomes critical in the so-called “developing” countries, as predictions state that over the next decades, new areas will be designated under multiple-use rather than under strict categories (McDonald & Boucher, 2011; Shafer, 2015).

Using biogeographical units as a basis to calculate representation has served to report conservation progress and to derive international policies (McNeely, Harrison & Dingwall, 1994). However, the proposed (gridded) physical approach diverges from biogeographical approaches and reveals new properties of protection and provides tools to explore nature representation at different spatial, temporal, and conceptual levels. First, the physical approach is sensitive to the estimation of the progress of conservation, since equality and extent are associated (Fig. 3B). Second, it allows mapping priorities at spatial resolutions which are only constrained by the available physical data. Third, it allows customizing the characterization scheme of “natural conditions” to any specific need. The selection of physical variables can be modified, expanded or improved with new or more suitable options. Fourth, it considers shifting physical patterns resulting from natural- or human-induced causes. Thus, geographical priorities could be forecasted under different climate scenarios (Scott, Malcolm & Lemieux, 2002; Theobald et al., 2015). At last, there are theoretical reasons and empirical evidence that show that physical variables are efficient estimators of the spatial distribution of species (Margules & Pressey, 2000). Thus, our physical approach undoubtedly complements traditional approaches based on biogeographical attributes as well as approaches dealing with the geographical gaps in biodiversity conservation, from species to ecosystems (Rodrigues et al., 2004).

## Conclusions

In this paper, we found that even the top national conservation networks are far from being saturated and balanced, as protected areas tend to be located in sparsely populated and isolated territories. We also found that the representation of natural conditions generally increases by expanding the extent of protected networks. However, temporal trends in conservation showed that equality is not gaining strength, contrary to the dominant conservation logic. Conservation priority maps can guide agencies in focusing their efforts, but demographic and economic limitations (e.g., agriculture, urban sprawl) are imposed on the deployment of new areas. In this sense, representation will only be strengthened if coupled with economic interests based on the provision of goods and services (including tourism).

##  Supplemental Information

10.7717/peerj.7155/supp-1 Figure S1 Correlation between physical variables Kendall’s correlation coefficients (*τ*) at national and continental levels; colors represents strength and sign of the correlation (from negative red, to white, to positive blue). Acronyms: PPT Precipitation, TMP Temperature, ELE Elevation, SLO Terrain slope, SOI Soil fertility.

10.7717/peerj.7155/supp-2 Figure S2 Alternative current representation Alternative relationship between current protection extent (%) and equality (G’) in South America. In contrast to Fig. 3, equality values (G’) were calculated considering a variable number of bins following the Sturges binning method (1926). Equality is calculated on the basis of physical continuous variables. Continental and Uruguayan results (in blue) were not used for linear regressions.

10.7717/peerj.7155/supp-3 Figure S3 Efficiency of increasing protection extent in the protection equality Calculated as the relationship of the differences between decades in extent and equality.

10.7717/peerj.7155/supp-4 Figure S4 Evolution of nature representation based on biogeographical units Temporal evolution of the relationship between protection extent (%) and equality (G’) in South America. Equality is calculated on the basis of biogeographical units (i.e., ecoregions). Each dot indicates the end point of a temporal period except for 1960 one, which indicates the data before 1960, inclusively. Uruguay has only one ecoregion (i.e. the Uruguayan savanna), and thus, no equality values could be quantified.

10.7717/peerj.7155/supp-5 Figure S5 Conservation priorities Detailed national conservation priorities (Pr) in South America according to the current spatial distribution of protection extent along physical gradients, classified into deciles (D). Red represents the highest priority, blue the lowest. White lines represent subnational political divisions. In the detail of South America, the entire continent is considered as a single unit of analysis .

10.7717/peerj.7155/supp-6 Figure S6 Distribution of protected areas Current spatial distribution of protection extent along physical gradients in South America. See graphic explanations in Fig. 2. Lower and upper *j* classes were grouped using the percentile values 0.025 and 0.975 of the *i* continuous variable. Red values indicate the equality of protection along each individual gradient according to the reverse of the Gini coefficient (G’).

10.7717/peerj.7155/supp-7 Table S1 Specific conservation priorities Main priorities in conservation at country and continental levels in South America considering protection extent along five physical gradients. Uruguay is excluded due to the very small extent of its protected area network (0.09% distributed in 4 units since 2006, Table 1).

10.7717/peerj.7155/supp-8 Supplemental Information 8 Raw data and code All tabular data, statistical tests, and figures are generated with the zipped files.

## Additional Information and Declarations

### Data Availability

The following information was supplied regarding data availability: All sources of information are depicted in the methods and in Table 2. Raw data and code are available in the Supplemental Files.
