# Supplementary figures and images for: Nature representation in South American protected areas: country contrasts and conservation priorities
Source: PeerJ. 2019 Jul 1;7:e7155. doi: 10.7717/peerj.7155 (PMC6611075; doi:10.7717/peerj.7155)

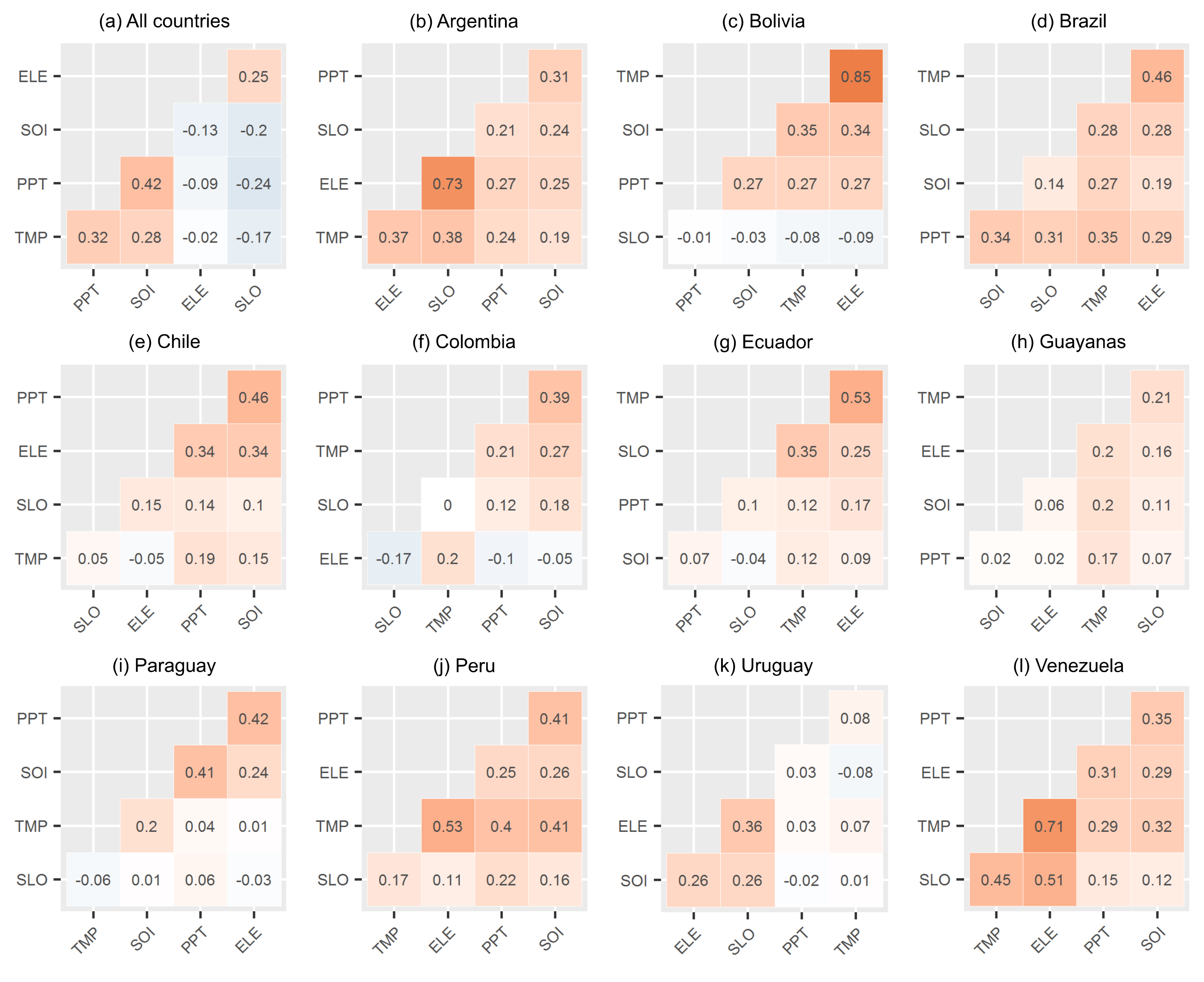

Supplement: Figure S1 — Kendall’s correlation coefficients (τ) at national and continental levels; colors represents strength and sign of the correlation (from negative red, to white, to positive blue). Acronyms: PPT Precipitation, TMP Temperature, ELE Elevation, SLO Terrain slope, SOI Soil fertility. [file peerj-07-7155-s001.png]

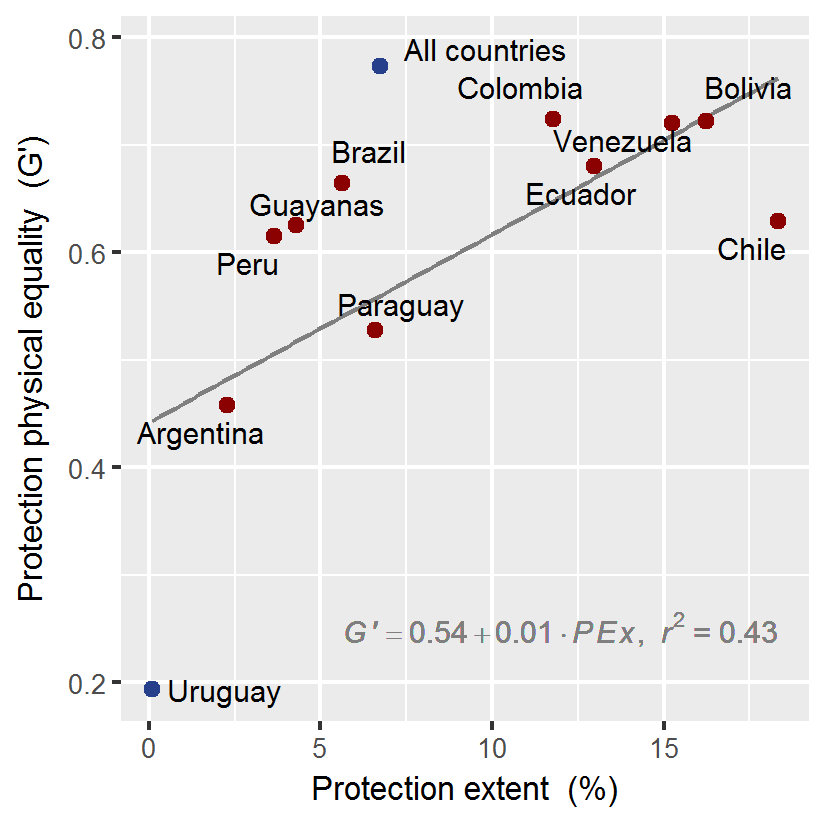

Supplement: Figure S2 — Alternative relationship between current protection extent (%) and equality (G’) in South America. In contrast to Fig. 3, equality values (G’) were calculated considering a variable number of bins following the Sturges binning method (1926). Equality is calculated on the basis of physical continuous variables. Continental and Uruguayan results (in blue) were not used for linear regressions. [file peerj-07-7155-s002.png]

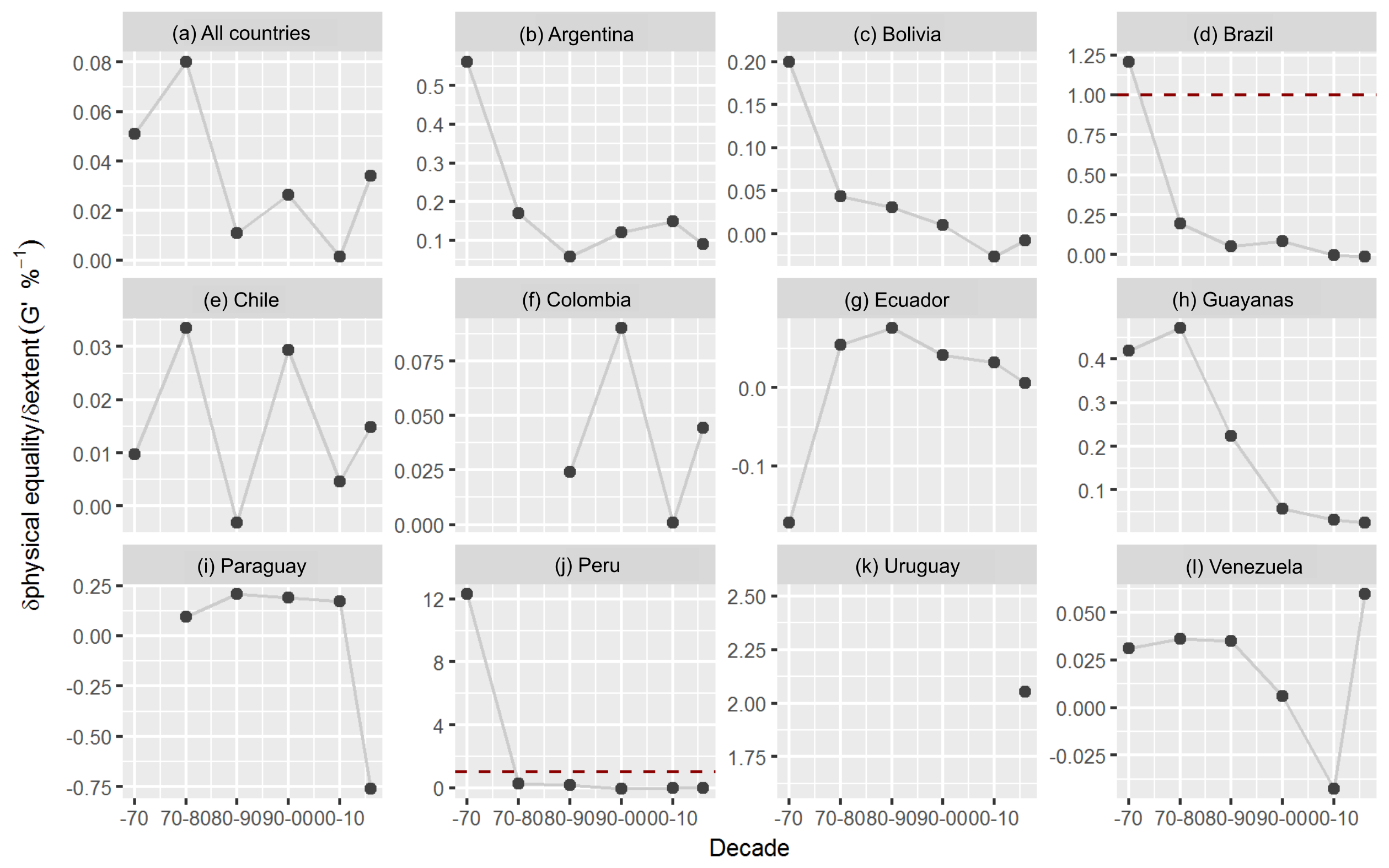

Supplement: Figure S3 — Calculated as the relationship of the differences between decades in extent and equality. [file peerj-07-7155-s003.png]

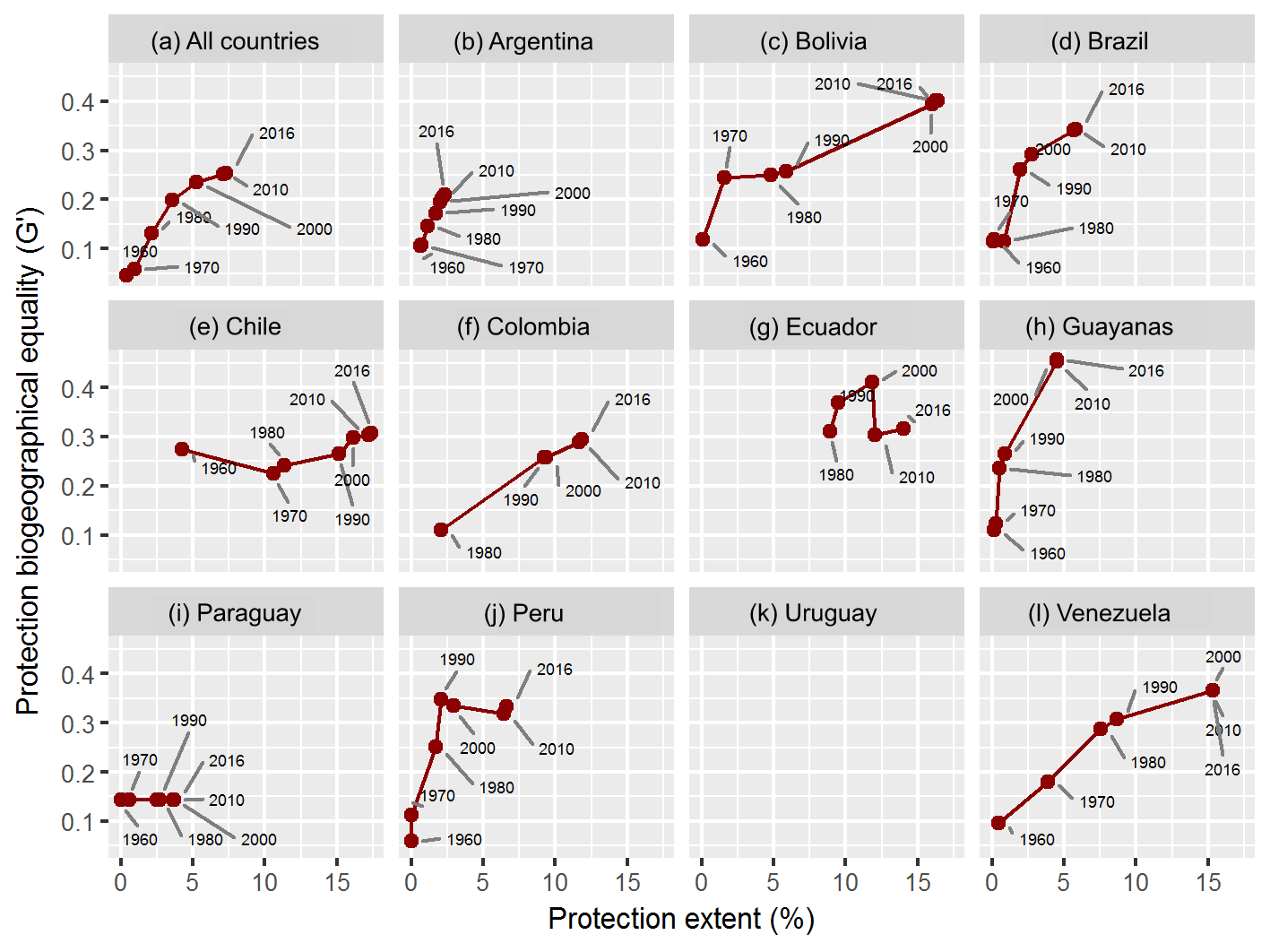

Supplement: Figure S4 — Temporal evolution of the relationship between protection extent (%) and equality (G’) in South America. Equality is calculated on the basis of biogeographical units (i.e., ecoregions). Each dot indicates the end point of a temporal period except for 1960 one, which indicates the data before 1960, inclusively. Uruguay has only one ecoregion (i.e. the Uruguayan savanna), and thus, no equality values could be quantified. [file peerj-07-7155-s004.png]

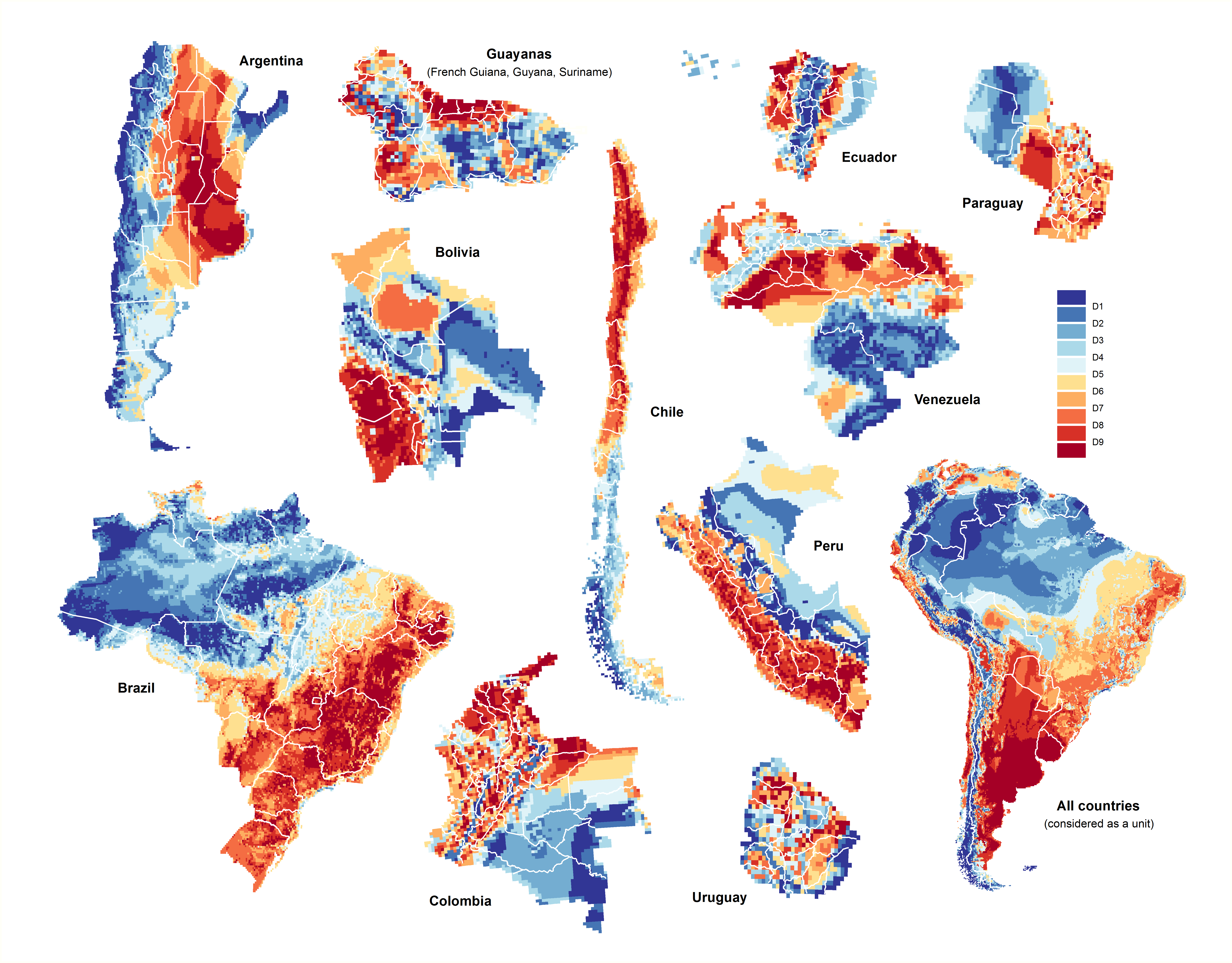

Supplement: Figure S5 — Detailed national conservation priorities (Pr) in South America according to the current spatial distribution of protection extent along physical gradients, classified into deciles (D). Red represents the highest priority, blue the lowest. White lines represent subnational political divisions. In the detail of South America, the entire continent is considered as a single unit of analysis . [file peerj-07-7155-s005.png]

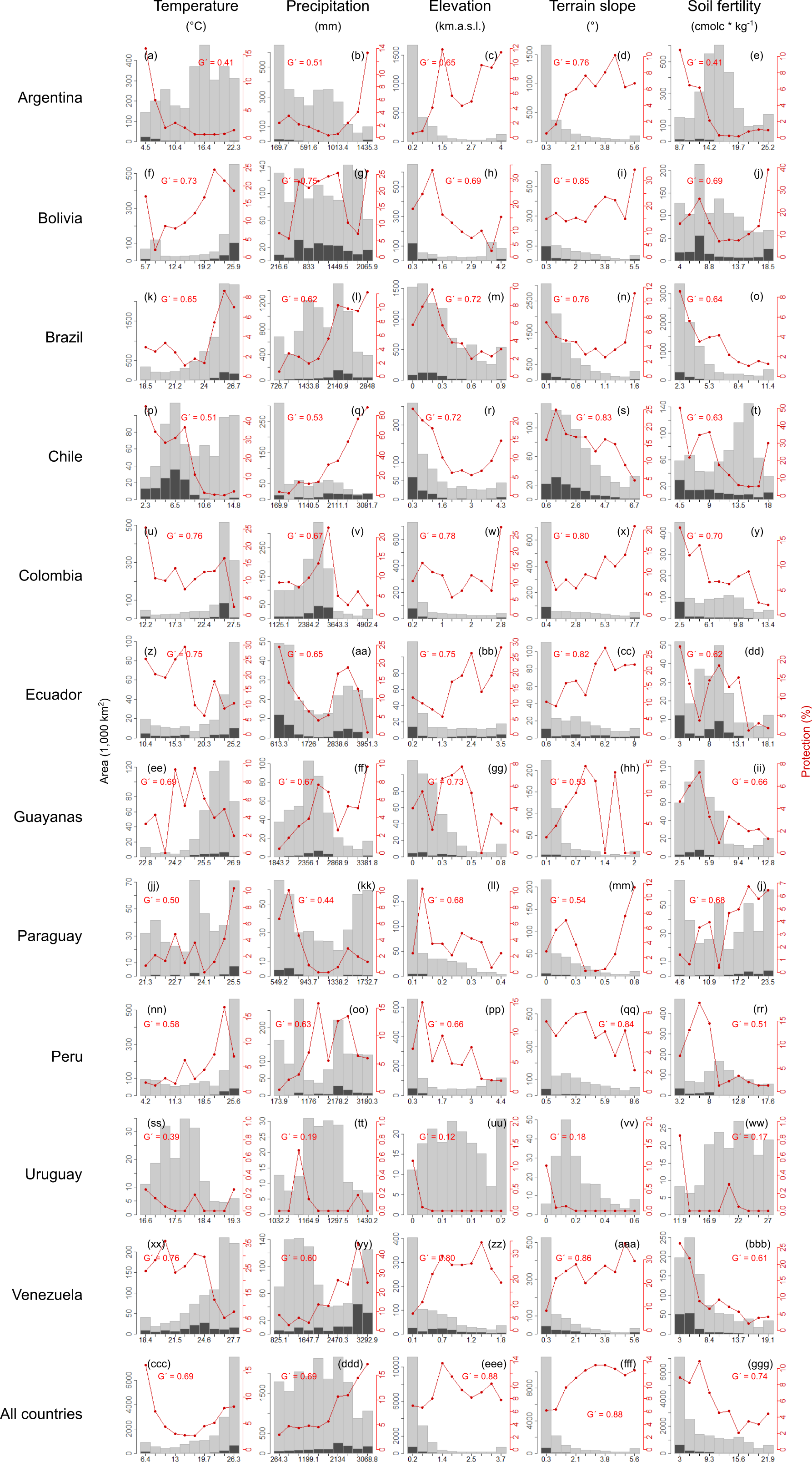

Supplement: Figure S6 — Current spatial distribution of protection extent along physical gradients in South America. See graphic explanations in Fig. 2. Lower and upper j classes were grouped using the percentile values 0.025 and 0.975 of the i continuous variable. Red values indicate the equality of protection along each individual gradient according to the reverse of the Gini coefficient (G’). [file peerj-07-7155-s006.png]
