# Supplementary material for: Nature representation in South American protected areas: country contrasts and conservation priorities
Source: PeerJ. 2019 Jul 1;7:e7155. doi: 10.7717/peerj.7155 (PMC6611075; doi:10.7717/peerj.7155)
Supplement: Table S1 — Main priorities in conservation at country and continental levels in South America considering protection extent along five physical gradients. Uruguay is excluded due to the very small extent of its protected area network (0.09% distributed in 4 units since 2006, Table 1). [file peerj-07-7155-s007.doc]

| **Country** | **Priorities** |
| --- | --- |
| Argentina | Humid, fertile lowlands of the Pampas ecoregion. Subhumid, warm temperate flat plains of the northern Espinal ecoregion, between the flooded wetlands along the [Paraná River](https://en.wikipedia.org/wiki/Paraná_River) and the [Sierras de Córdoba](https://en.wikipedia.org/wiki/Sierras_de_Córdoba) mountain range. Semiarid, hot and fertile [lowland](https://en.wikipedia.org/wiki/Lowland)s of the central and northern Dry Chaco ecoregion. |
| Bolivia | Arid to semiarid, cold plateaus of the Puna ecoregions. Humid lowlands plains of the southwestern Amazon basin (Beni savanna ecoregion). |
| Brazil | Semiarid, hot, inland areas of northeastern Brazil (Caatinga ecoregion). Inland areas from the Bahia coastal forests (Bahia interior forests ecoregion). Eastern, central and southern subhumid highlands of the Cerrado ecoregion. Middle-level plateaus to high slope mountain areas of southern Atlantic Brazil (Araucaria moist forests ecoregion). Tropical dry forests of eastern Brazil, between the Caatinga and the Cerrado ecoregions (Atlantic dry forest ecoregion). Fertile plains of the southern part of the Rio Grande do Sul state (Uruguayan savanna ecoregion). |
| Colombia | Northern hot, semiarid to subhumid plains (including dry and moist forests and xeric shrublands ecoregions). Northern strip of the Llanos ecoregion, characterized by hot, subhumid to humid conditions, and surrounding broadleaf forests. Western coast of the country, between the Pacific Ocean and the peaks of the northern Andes (Chocó-Darién moist forests and Northwestern Andean montane forests ecoregions). Fertile central valley of the upper Magdalena River, between the [eastern](https://en.wikipedia.org/wiki/Cordillera_Oriental_(Colombia)) and [central ranges](https://en.wikipedia.org/wiki/Cordillera_Central_(Colombia)) of the Andes (Magdalena Valley dry forests ecoregion), and higher lands on both sides of the valley (Magdalena Valley montane forests ecoregion). Dry to highly humid, fertile regions of the Cauca Valley, between the central and western ranges of the Andes (Cauca Valley montane and dry forests ecoregions). |
| Chile | North half of the country, characterized by temperate to warm, semiarid to arid conditions (particularly the Chilean matorral and Atacama desert ecoregions). Semiarid, cold to temperate, eastern foothills of the Southern Andes (Patagonian steppe ecoregion). |
| Ecuador | Warm and fertile central coasts (Ecuadorian dry forests ecoregion). Humid, western plains and foothills of the [Andes](https://en.wikipedia.org/wiki/Andes) (Western Ecuador moist forests ecoregion). Western strip of the Napo moist forest ecoregion. |
| Guayanas | Coastal areas of Surinam and Guyana, and the southern driest portion of Guyana (Guianan savanna ecoregion). Contrary to its neighbors, this last country has a very low protection extent (Table 1). |
| Paraguay | More humid and relatively colder central and eastern half of the country (Humid Chaco and Atlantic forest ecoregions). |
| Peru | Arid coastal plains of the Sechura desert ecoregion. Arid to semiarid, cold plateaus of the Puna ecoregions. Humid eastern slopes of the Peruvian Andes (Peruvian Yungas ecoregion). |
| Venezuela | Northern and northeastern Llanos ecoregion, characterized by hot, subhumid to humid conditions, and surrounding broadleaf forests and xeric shrublands (Apure-Villavicencio dry forests and La Costa xeric shrublands ecoregions). Fertile, hot plains of West Lake Maracaibo (Maracaibo dry forests ecoregion) and the hot plains of the northeastern states of Monagas and Delta Amacuro (Orinoco wetlands and Orinoco Delta swamp forests ecoregions). |
